# Socioeconomic disparities in abdominal aortic aneurysm repair rates and survival

**DOI:** 10.1093/bjs/znac222

**Published:** 2022-08-11

**Authors:** Ravi Maheswaran, Thaison Tong, Jonathan Michaels, Paul Brindley, Stephen Walters, Shah Nawaz

**Affiliations:** Epidemiology and Public Health, School of Health and Related Research, University of Sheffield, Sheffield, UK; School of Health and Related Research, University of Sheffield, UK; Clinical Decision Science, School of Health and Related Research, University of Sheffield, UK; Department of Landscape Architecture, University of Sheffield, Sheffield, UK; Medical Statistics and Clinical Trials, School of Health and Related Research, University of Sheffield, Sheffield, UK; Sheffield Vascular Institute, Sheffield Teaching Hospitals NHS Foundation Trust, UK

## Abstract

**Background:**

Abdominal aortic aneurysm (AAA) is more prevalent in socioeconomically disadvantaged areas. This study investigated socioeconomic disparities in AAA repair rates and survival.

**Methods:**

The study used ecological and cohort study designs, from 31 672 census areas in England (April 2006 to March 2018), the Index of Multiple Deprivation 2010 as the area-level deprivation indicator, and Poisson, logistic and Cox regression.

**Results:**

Some 77 606 patients (83.4 per cent men) in four age categories (55–64, 65–74, 75–84, 85 or more years) were admitted with AAA from a population aged at least 55 years of 14.7 million. Elective open and endovascular repair rates were 41 (95 per cent c.i. 23 to 61) and 60 (36 to 89) per cent higher respectively among men aged 55–64 years in the most *versus* least deprived areas by quintile. This differences diminished and appeared to reverse with increasing age, with 26 (−1 to 45) and 25 (13 to 35) per cent lower rates respectively in men aged 85 years or more in the most deprived areas. Men admitted from more deprived areas were more likely to die in hospital without aneurysm repair. Among those who had aneurysm repair, this was more likely to be for a ruptured aneurysm than among men from less deprived areas. For intact aneurysm repair, they were relatively more likely to have this during an emergency admission. The mortality rate after repair was higher for men from more deprived areas, although the hazard diminished with age. Patterns were unclear for women.

**Conclusion:**

There were clear socioeconomic disparities in operation rates, mode of presentation, and outcome for AAA surgery. Policies are needed to address these disparities.

## Teaser

Abdominal aortic aneurysm (AAA) is more prevalent in socioeconomically disadvantaged areas. This study found clear socioeconomic disparities in relation to operation rates, mode of presentation, method of treatment, and outcome of AAA surgery. Policies are needed to address these disparities. R. Maheswaran *et al*.

## Introduction

Abdominal aortic aneurysm (AAA) has prevalence estimates in the region of 1.2–7.6 per cent depending on the population screened^1–6^. Rupture of AAA is life-threatening and a meta-analysis^7^estimated that the population-based mortality rate following rupture was 81 per cent. Socioeconomic inequalities in health exist in numerous countries^8,9^. These socioeconomic inequalities are reflected in the prevalence of AAA in England based on data from the national AAA screening programme for men aged 65 years; the prevalence in the most deprived areas by quintile is 80 per cent higher than that in the least deprived areas^5^.

Disparities in surgical access and outcomes exist for many types of surgery. However, the association between socioeconomic disadvantage and population-based AAA repair rates has not been examined. A few studies^10–13^ have examined mode of presentation in relation to socioeconomic deprivation. Ruptured AAA may be becoming less common. However, a significant proportion of patients presenting with ruptured aneurysm are not operated on and the non-intervention rate is much higher among older people^10,11,13^. Some studies suggest that patients from socioeconomically disadvantaged backgrounds may be more likely to present with ruptured AAA^10,14–16^. However, it is not known whether the non-intervention rate is higher in patients from disadvantaged areas.

Among those who undergo aneurysm repair, it is unclear whether socioeconomically disadvantaged patients are more likely to have this done as an emergency procedure^17,18^. Although the use of endovascular aneurysm repair (EVAR) has increased substantially over the past 15–20 years^10,11,13,19,20^, the evidence linking socioeconomic disadvantage and method of repair is mixed^18,19,21–23^. The much less invasive nature of EVAR means that it is increasingly being used in higher-risk patients, including older patients in whom AAA is considerably more prevalent^13,20^. However, variation in the use of EVAR in relation to socioeconomic disadvantage in the older population has not been investigated. Survival after AAA repair may be worse in patients experiencing socioeconomic disadvantage^10,14,22,24–26^, although this has not been found in other studies^16,18,23,27^.

Healthcare funding systems play a major role in determining access to healthcare^28^. The aim of this national population-based study was to examine socioeconomic variation in AAA repair rates and outcomes by age and also sex, as studies^29,30^ have reported lower intervention rates and worse outcomes for women.

## Methods

### Study design, area, and socioeconomic deprivation

A population-based (ecological) study design, with data at a fine geographical scale, was used to examine AAA repair rates, and a cohort study design to examine survival after surgery. Lower layer super output areas (LSOAs) in England were used as the basic geographical units^31^. LSOAs are census areas defined in the 2001 national census and each contains approximately 1500 people. There were changes to a small number of LSOAs in the 2011 census. To maintain consistent geography across the study time span (2006–2018), the analysis was restricted to 31 672 (96.4 per cent) of the 32 844 LSOAs in 2011 with unchanged boundaries. Data on men and women aged 55 years or more were examined using LSOA mid-year population estimates by 10-year age band (55–64 to 85 or more years).

The Income Domain from the Index of Multiple Deprivation (IMD) 2010 was used as the indicator of socioeconomic deprivation at the LSOA level^32^. The IMD is the national index of deprivation used widely by government agencies in England. Use of an IMD from a single year allowed a consistent set of LSOAs (and therefore consistent geography) to be maintained in each deprivation category across the time span.

### Data on hospital admissions and mortality

Hospital Episode Statistics (HES) data on admissions to National Health Service (NHS) hospitals in England from April 2006 to March 2018 were examined; data are provided in financial years, which run from 1 April to 31 March the following year. Admissions for repair of infrarenal AAA were examined using OPCS procedure codes, the standard classification system used by the NHS in England^33^. All admissions for an individual patient were identified using a pseudoanonymized patient identifier. Patients were then classified using the index admission, which was defined as the admission in which patients received their first AAA repair. Patients admitted with an AAA who died in hospital without repair were also examined (described below).

Patients who had surgery were grouped into four categories based on the operative procedure during the index admission: elective (planned) open repair; elective EVAR; emergency repair of non-ruptured aneurysm; and emergency repair of ruptured aneurysm. The latter two categories were not subdivided into open and EVAR procedures as the procedure rates became quite low. A fifth category comprised patients admitted with an AAA who had no repair and died in hospital during the same admission. These patients had a primary diagnosis of AAA and a treatment or main specialty code of general or vascular surgery. The definition was arrived at after examination of sample records of deaths in hospital; full details are provided elsewhere^34^.

Admissions data were obtained from NHS Digital, the national organization that manages all NHS hospital admissions data in England. The data provided included linked mortality records containing date and cause of death for admitted patients who subsequently died during the period to 31 March 2018. The admissions data also contained information on co-morbidities recorded using ICD-10 codes. Information on co-morbidities was obtained from the index admission record and from any admission in the previous 3 months. Eight conditions were considered as co-morbidities (see results). These were based on the Royal College of Surgeons Charlson Score^35^, but modified by the expert clinical advisory panel for this project.

### Statistical analysis

Population-based AAA rates were analysed using Poisson regression, with confidence intervals inflated to take account of any overdispersion. The odds of having an aneurysm repair after admission, of in-hospital death following aneurysm repair, and of readmission within 30 days of discharge were examined using logistic regression. Duration of hospital stay after surgery was evaluated using generalized linear regression with a log link because of the positive skew in the data. Survival after aneurysm repair was analysed using Cox proportional hazards modelling. All models were adjusted for year of admission. Logistic, linear, and Cox regression models were also adjusted for co-morbidities (included as 8 separate categorical variables).

Because data were very sparse at the LSOA level, LSOAs were grouped into five categories using deprivation quintiles, and the median deprivation value in each category was used as a continuous variable in the statistical analyses. Rate ratios, ORs, and HRs were calculated as a trend across all quintile categories and presented with 95 per cent confidence intervals for the most relative to the least deprived category.

Supplementary analyses examined in-hospital death, duration of hospital stay, and readmissions after elective AAA repair, hospital location deprivation levels, hospital AAA repair volumes, and causes of death following AAA repair.

## Results

### Patient characteristics

Some 77 606 patients aged at least 55 years were admitted to hospital with AAA over the 12-year study interval, with a corresponding average denominator population of 14.7 million (*Table 1*). Some 83.4 per cent were men and 42.6 per cent were in the 75–84-year age group; 68 360 patients (88.1 per cent) had an AAA repair, whereas 9246 patients (11.9 per cent) did not have the aneurysm repaired and died in hospital during the same admission. The majority of admissions (61.8 per cent) were for elective (planned) operations. Of the eight co-morbidities examined, coronary heart disease and chronic obstructive pulmonary disease were the most prevalent, with 24.9 and 24.4 per cent of patients respectively having these conditions.

**Table 1:** Characteristics of patients admitted for abdominal aortic aneurysm repair in England (April 2006 to March 2018)

	Men (*n* = 64 742)	Women (*n* = 12 864)	All (*n* = 77 606)
**Age (years)**			
55–64	6450 (10.0)	605 (4.7)	7055 (9.1)
65–74	24 595 (38.0)	3191 (24.8)	27 786 (35.8)
75–84	26 979 (41.7)	6045 (47.0)	33 024 (42.6)
≥ 85	6718 (10.4)	3023 (23.5)	9741 (12.6)
**Procedure**			
Elective open repair	17 639 (27.2)	2831 (22.0)	20 470 (26.4)
Elective EVAR	24 455 (37.8)	3026 (23.5)	27 481 (35.4)
Emergency repair of non-ruptured aneurysm	6837 (10.6)	1464 (11.4)	8301 (10.7)
Repair of ruptured aneurysm	10 054 (15.5)	2054 (16.0)	12 108 (15.6)
In-hospital death without aneurysm repair	5757 (8.9)	3489 (27.1)	9246 (11.9)
Co-morbidities*			
Coronary artery disease	16 583 (25.6)	2718 (21.1)	19 301 (24.9)
Heart failure	3965 (6.1)	901 (7.0)	4866 (6.3)
Cerebrovascular disease	3326 (5.1)	778 (6.0)	4104 (5.3)
Chronic obstructive pulmonary disease	15 286 (23.6)	3644 (28.3)	18 930 (24.4)
Diabetes	9578 (14.8)	1463 (11.4)	11 041 (14.2)
Renal disease	4517 (7.0)	1092 (8.5)	5609 (7.2)
Cancer	7094 (11.0)	771 (6.0)	7865 (10.1)
Moderate or severe liver disease	383 (0.6)	76 (0.6)	459 (0.6)

Values are n (% by column), except *n (% by condition). EVAR, endovascular aneurysm repair.

**Fig. 1:**
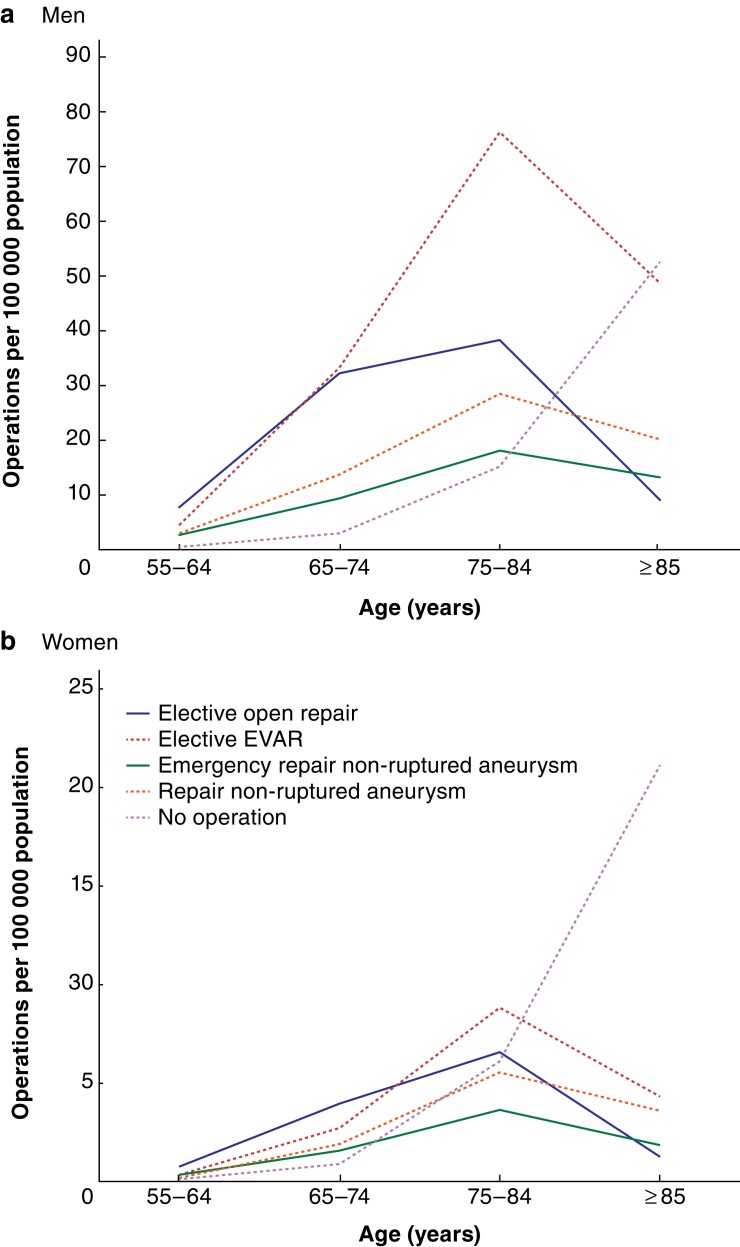
Average annual operative procedure rates for abdominal aortic aneurysm repair by age, sex, and type of procedure in England (April 2006 to March 2018) **a** Men and **b** women. EVAR, endovascular aneurysm repair.

Rates of surgery were generally much higher in men (*Fig. 1*). Procedure rates increased with age and peaked in the 75–84-year age group. The rate of death in hospital without AAA repair rose with increasing age, but was substantially higher among patients aged 85 years or more. The percentage of women admitted with an AAA who died in hospital without aneurysm repair was higher than that for men in all age groups (*Table 2*). Among patients aged 85 years or more, 36.5 per cent of men admitted died without aneurysm repair compared with 65.8 per cent of women. General trends over time in population-based rates, and general patterns in survival curves following AAA repair, are described in the *Supplementary material*.

**Fig. 2:**
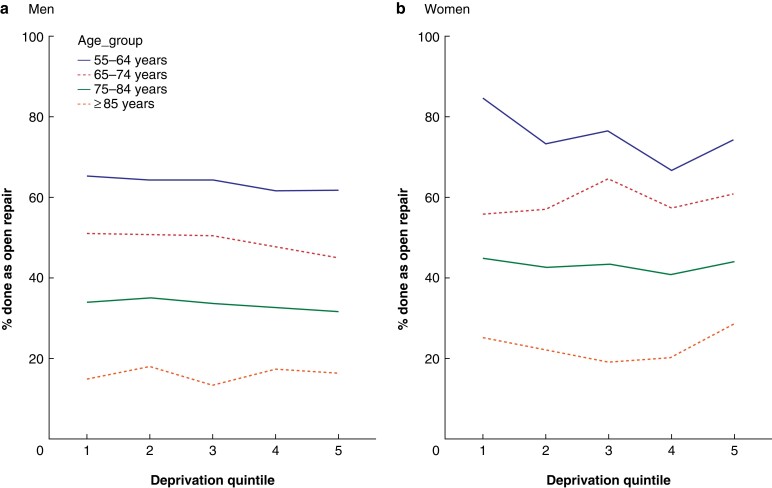
Percentage of all elective abdominal aortic aneurysm repairs (open and endovascular) that were done as open procedures, by socioeconomic deprivation category, sex, and age in England (April 2006 to March 2018) **a** Men and **b** women. Deprivation quintiles: 1, least deprived; 5, most deprived.

**Table 2:** **Percentage of patients admitted with an abdominal aortic aneurysm who died in hospital without aneurysm repair in England (April 2006 to March 2018**)

	Admitted and died without AAA repair	
Age (years)	Men	Women
**55–­64**	160 (2.5)	39 (6.4)
**65–74**	825 (3.4)	256 (8.0)
**75–84**	2318 (8.6)	1204 (19.9)
**≥ 85**	2454 (36.5)	1990 (65.8)

Values are *n* (%). AAA, abdominal aortic aneurysm.

### Socioeconomic deprivation and population-based aneurysm repair rate ratios

Among men aged 55–64 years, the elective open repair rate was 41 (95 per cent c.i. 23 to 61) per cent higher in the most deprived areas (*Table 3*). However, this pattern not only diminished, but reversed in the groups aged 75–84 and 85 or more years, with elective open repair rates 22 (13 to 30) and 26 (−1 to 45) per cent lower respectively in the most deprived areas. These rate ratios could not be adjusted for co-morbidities as the denominator populations did not contain the required co-morbidity information.

**Table 3:** Rate ratios for abdominal aortic aneurysm repair in the most relative to the least socioeconomically deprived quintile category by age, sex, and type of procedure in England (April 2006 to March 2018)

		Men		Women	
	Age (years)	*n*	Rate ratio	*n*	Rate ratio
**Elective open repair**	55–64	2718	1.41 (1.23, 1.61)	272	2.24 (1.63, 3.08)
	65–74	8642	1.00 (0.92, 1.09)	1147	2.04 (1.68, 2.48)
	75–84	5856	0.78 (0.70, 0.87)	1295	1.31 (1.12, 1.54)
	≥ 85	423	0.74 (0.55, 1.01)	117	1.63 (0.95, 2.78)
**Elective EVAR**	55–64	1577	1.60 (1.36, 1.89)	94	2.91 (1.65, 5.13)
	65–74	8929	1.33 (1.23, 1.44)	788	1.96 (1.61, 2.39)
	75–84	11 675	0.95 (0.90, 1.01)	1739	1.48 (1.27, 1.72)
	≥ 85	2274	0.75 (0.65, 0.87)	405	1.31 (0.99, 1.75)
**Emergency repair of non-ruptured aneurysm**	55–64	936	1.86 (1.56, 2.21)	124	2.98 (1.80, 4.92)
	65–74	2514	1.50 (1.31, 1.73)	452	2.15 (1.62, 2.85)
	75–84	2769	1.10 (0.98, 1.24)	714	1.79 (1.43, 2.24)
	≥ 85	618	1.03 (0.80, 1.32)	174	1.69 (1.10, 2.59)
**Repair of ruptured aneurysm**	55–64	1059	1.70 (1.42, 2.03)	76	1.80 (0.86, 3.79)
	65–74	3685	1.47 (1.31, 1.65)	548	2.48 (1.99, 3.11)
	75–84	4361	1.16 (1.05, 1.28)	1093	1.86 (1.57, 2.21)
	≥ 85	949	0.90 (0.73, 1.10)	337	1.23 (0.90, 1.69)
**No operation**	55–64	160	2.30 (1.52, 3.48)	39	1.75 (0.67, 4.57)
	65–74	825	2.28 (1.81, 2.88)	256	2.90 (1.99, 4.23)
	75–84	2318	1.52 (1.33, 1.75)	1204	2.26 (1.91, 2.68)
	≥ 85	2454	1.12 (0.98, 1.27)	1990	1.29 (1.13, 1.46)

Values in parentheses are 95 per cent confidence intervals. Rate ratios calculated as a trend across all quintile categories, adjusted for year, and expressed as the ratio for the most relative to the least deprived category. EVAR, endovascular aneurysm repair.

A similar pattern was seen in elective EVAR rates, for which the rate was 60 (36 to 89) per cent higher for men in the most deprived areas in the 55–64-year age group but diminished and reversed with increasing age, resulting in a 25 (13 to 35) per cent lower rate in the most deprived areas among patients aged at least 85 years.

The emergency repair rate for non-ruptured AAAs was 86 (56 to 121) per cent higher in men in the most deprived areas in the 55–64-year age group. This difference diminished with increasing age but did not appear to reverse.

The repair rate for ruptured AAA was 70 (42 to 103) per cent higher among men in the most deprived areas in the 55–64-year age group but diminished with increasing age, and was 10 (−10 to 27) per cent lower in the most deprived areas among patients aged 85 years or older.

Death rates for men who were admitted and died in hospital with no AAA repair were 130 (52 to 248) per cent higher in the most deprived areas in the 55–64-year age group. Although this excess diminished with increasing age, it generally remained noticeably higher in the older age groups.

The patterns were somewhat different in women. The ratio of rates in the most relative to the least deprived areas was generally higher than in men in all procedure categories and age groups. The reduction in rate ratios with increasing age was less consistent and less marked. In particular, there was no reversal in rate ratios for elective repair in the older age groups.

### Socioeconomic deprivation and aneurysm repair following admission

The prevalence of co-morbidities increased with increasing deprivation (*Table 4*).

**Table 4:** Co-morbidities in patients admitted with an abdominal aortic aneursym by socioeconomic deprivation quintile category in England (April 2006 to March 2018)

	Socioeconomic deprivation category†					
	1 (*n* = 15 073)	2 (*n* =17 337)	3 (*n* = 17 163)	4 (*n* = 15 474)	5 (*n* = 12 559)	Adjusted OR*
**Coronary artery disease**	3593 (23.8)	4095 (23.6)	4158 (24.2)	3959 (25.6)	3496 (27.8)	1.30 (1.24, 1.37)
**Heart failure**	887 (5.9)	1032 (6.0)	1028 (6.0)	1019 (6.6)	900 (7.2)	1.30 (1.20, 1.42)
**Cerebrovascular disease**	741 (4.9)	872 (5.0)	903 (5.3)	855 (5.5)	733 (5.8)	1.23 (1.13, 1.35)
**Chronic obstructive pulmonary disease**	3050 (20.2)	3795 (21.9)	4015 (23.4)	4129 (26.7)	3941 (31.4)	1.80 (1.72, 1.89)
**Diabetes**	1959 (13.0)	2276 (13.1)	2422 (14.1)	2336 (15.1)	2048 (16.3)	1.36 (1.28, 1.44)
**Renal disease**	991 (6.6)	1130 (6.5)	1265 (7.4)	1190 (7.7)	1033 (8.2)	1.38 (1.27, 1.49)
**Cancer**	1573 (10.4)	1807 (10.4)	1666 (9.7)	1556 (10.1)	1263 (10.1)	1.05 (0.98, 1.13)
**Moderate or severe liver disease**	103 (0.7)	86 (0.5)	88 (0.5)	86 (0.6)	96 (0.8)	1.27 (0.97, 1.66)

Values are n (%) unless otherwise indicated; *values in parentheses are 95 per cent confidence intervals. †Deprivation quintiles: 1, least deprived; 5, most deprived. ORs calculated as a trend across all categories, adjusted for age and sex, and expressed as the ratio for the most relative to the least deprived category .

Men admitted from the most deprived areas had higher odds of the admission ending in death in hospital without AAA repair in all age bands examined (*Table 5*). Among men who had AAA repair, the odds of this being performed for a ruptured aneurysm were higher in men admitted from the most deprived areas. Among men who had repair of an intact AAA, men admitted from the most deprived areas had higher odds of this being performed as an emergency procedure. In contrast, among men who had elective AAA repair, men from the most deprived areas had lower odds of elective procedures being carried out as open repairs, although this was clearly seen only in the 65–74- and 75–84-year age groups (*Table 5* and *Fig. 2*). Adjustment for co-morbidities made little difference to the ORs (*Table 5*).

**Table 5:** ORs for the most relative to the least socioeconomically deprived quintile category for proportion of admissions ending in death in hospital without abdominal aortic aneurysm (AAA) repair, proportion of all AAA repairs that were for ruptured aneurysms, proportion of all repairs for intact AAA that were carried out as emergency admissions, and proportion of all elective AAA repairs that were carried out as open repairs, in England (April 2006 to March 2018)

	OR			
	Men		Women	
Age (years)	Adjusted for year	Adjusted for year and co-morbidities	Adjusted for year	Adjusted for year and co-morbidities
**Admission ending in death in hospital without AAA repair**				
55–64	1.51 (0.98, 2.32)	1.42 (0.92, 2.20)	0.68 (0.26, 1.74)	0.74 (0.27, 2.01)
65–74	1.95 (1.61, 2.36)	1.75 (1.44, 2.13)	1.40 (0.99, 1.98)	1.29 (0.90, 1.85)
75–84	1.60 (1.42, 1.82)	1.47 (1.30, 1.67)	1.50 (1.26, 1.79)	1.37 (1.14, 1.64)
≥ 85	1.35 (1.15, 1.58)	1.28 (1.09, 1.50)	0.94 (0.75, 1.18)	0.94 (0.75, 1.18)
**Repair of ruptured (relative to intact) aneurysms**				
55–64	1.11 (0.92, 1.34)	1.20 (0.99, 1.46)	0.72 (0.36, 1.45)	0.85 (0.42, 1.73)
65–74	1.26 (1.13, 1.39)	1.31 (1.18, 1.46)	1.24 (0.96, 1.60)	1.20 (0.92, 1.55)
75–84	1.27 (1.15, 1.40)	1.31 (1.18, 1.44)	1.28 (1.06, 1.55)	1.34 (1.10, 1.62)
≥ 85	1.11 (0.88, 1.40)	1.16 (0.92, 1.47)	0.86 (0.58, 1.27)	0.85 (0.57, 1.27)
**Surgery for intact aneurysms being carried out as an emergency admission**				
55–64	1.30 (1.06, 1.59)	1.33 (1.09, 1.62)	1.31 (0.74, 2.31)	1.39 (0.77, 2.51)
65–74	1.33 (1.17, 1.50)	1.34 (1.18, 1.51)	1.05 (0.78, 1.39)	1.03 (0.77, 1.37)
75–84	1.23 (1.09, 1.39)	1.25 (1.10, 1.41)	1.27 (1.01, 1.60)	1.24 (0.98, 1.57)
≥ 85	1.37 (1.04, 1.80)	1.40 (1.06, 1.85)	1.21 (0.74, 2.00)	1.16 (0.70, 1.93)
**Elective procedures being carried out as open repairs**				
55–64	0.90 (0.75, 1.08)	1.01 (0.84, 1.22)	0.83 (0.43, 1.62)	0.78 (0.40, 1.53)
65–74	0.77 (0.71, 0.85)	0.84 (0.76, 0.92)	1.04 (0.79, 1.36)	1.09 (0.83, 1.45)
75–84	0.79 (0.71, 0.88)	0.83 (0.74, 0.92)	0.94 (0.75, 1.18)	0.96 (0.76, 1.21)
≥ 85	0.89 (0.61, 1.28)	0.90 (0.62, 1.31)	1.18 (0.62, 2.25)	1.13 (0.58, 2.18)

Values in parentheses are 95 per cent confidence intervals. ORs calculated as a trend across all quintile categories and expressed as the ratio for the most relative to the least deprived category. AAA, abdominal aortic aneurysm.

In women, however, there were no clear patterns of association between socioeconomic deprivation and the odds of repair after admission.

### Socioeconomic deprivation and survival following aneurysm repair

**Fig. 3:**
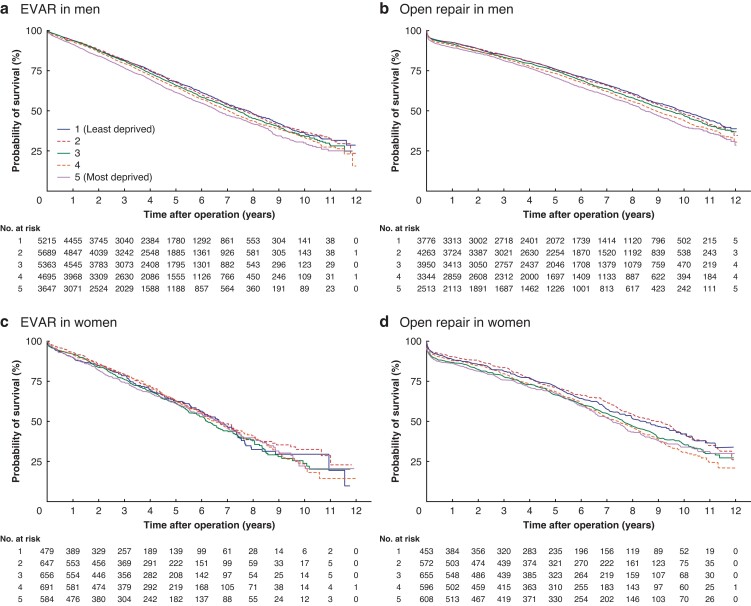
Survival after elective endovascular aneurysm repair or elective open repair by socioeconomic deprivation category and sex in England (April 2006 to March 2018) **a** Endovascular aneurysm repair (EVAR) in men, **b** open repair in men, **c** EVAR in women, and **d** open repair in women.

Unadjusted survival rates after elective EVAR and elective open surgery were lower in men from more socioeconomically deprived areas (*Fig. 3*). HRs for death after AAA repair, adjusted for co-morbidities, were generally higher in men from the most deprived areas for all procedure types (*Table 6*). However, the magnitude of the increase in HRs diminished, albeit inconsistently, with increasing age. In women, confidence intervals were wide as death counts were low and there were no clear patterns of association with socioeconomic deprivation after aneurysm repair.

**Table 6:** Adjusted hazard ratios for death following abdominal aortic aneurysm repair in the most relative to the least socioeconomically deprived quintile category by age, sex, and type of procedure in England (April 2006 to March 2018)

		Men		Women	
	Age (years)	No. of deaths	HR	No. of deaths	HR
**Elective open repair**	55–64	570	1.28 (1.05, 1.56)	78	1.03 (0.60, 1.78)
	65–74	2680	1.32 (1.19, 1.46)	444	1.20 (0.96, 1.50)
	75–84	3102	1.18 (1.07, 1.30)	755	1.28 (1.06, 1.53)
	≥ 85	313	1.16 (0.82, 1.66)	81	0.72 (0.40, 1.29)
**Elective EVAR**	55–64	337	1.49 (1.15, 1.92)	19	0.44 (0.10, 1.94)
	65–74	2329	1.41 (1.28, 1.56)	256	1.57 (1.17, 2.09)
	75–84	4678	1.21 (1.12, 1.31)	716	0.85 (0.70, 1.03)
	≥ 85	1219	1.02 (0.86, 1.21)	207	0.82 (0.55, 1.23)
**Emergency repair of non-ruptured aneurysm**	55–64	267	1.41 (1.07, 1.86)	49	0.71 (0.34, 1.49)
	65–74	1003	1.24 (1.07, 1.44)	219	1.18 (0.85, 1.64)
	75–84	1494	1.16 (1.01, 1.34)	415	1.17 (0.93, 1.48)
	≥ 85	408	1.34 (1.03, 1.75)	117	1.31 (0.81, 2.13)
**Repair of ruptured aneurysm**	55–64	409	1.22 (0.97, 1.52)	38	0.80 (0.32, 2.01)
	65–74	1942	1.28 (1.14, 1.44)	349	1.29 (0.99, 1.66)
	75–84	3134	1.09 (0.99, 1.20)	843	1.17 (1.00, 1.37)
	≥ 85	774	0.94 (0.76, 1.17)	283	1.39 (0.99, 1.93)

Values in parentheses are 95 per cent confidence intervals. HRs calculated as a trend across all quintile categories, adjusted for year and co-morbidities, and expressed as the ratio for the most relative to the least deprived category. EVAR, endovascular aneurysm repair.

There was no evidence of a gradient in association between the number of hospitals located in each deprivation category area, or the percentage of these hospitals with an annual AAA surgery volume of at least 60, and the deprivation level of the area (*Figs S1–S4* and *Tables S1–S5*). The percentage of patients undergoing elective AAA repair in each deprivation category who had the procedure performed at a hospital with an annual volume of at least 60 was generally similar across deprivation categories. Patients living in more socioeconomically deprived areas had higher odds of in-hospital death, longer duration of hospital stay, and higher odds of readmission after elective AAA surgery compared with patients living in less deprived areas. Patients treated in hospitals located in more deprived areas had a longer duration of stay than those treated in hospitals in less deprived areas. However, there was no evidence of an association between the deprivation level of the area in which the hospital was located and in-hospital mortality or readmissions after elective AAA surgery.

## Discussion

Although elective open repair and elective EVAR rates were higher in younger men living in more, compared with less, socioeconomically deprived areas, the difference not only diminished with increasing age but reversed, resulting in lower elective repair rates in older men in more deprived areas. There were higher emergency and rupture repair rates in more deprived areas which diminished with increasing age. Population-based non-intervention rates (death rates based on patients who were admitted and died in hospital without aneurysm repair) were higher in more deprived areas. Although this excess diminished with increasing age, it generally remained noticeably higher in more deprived areas in the older age groups.

Among admitted patients, men from more deprived areas were more likely to die in hospital without having aneurysm repair than men from less deprived areas. Among men who had aneurysm repair, this was relatively more likely to be for a ruptured aneurysm in more deprived compared with less deprived areas. Amongst men who had intact aneurysm repair, those from more deprived areas were more likely to have this carried out as an emergency procedure than men from less deprived areas. Mortality after repair was higher for all procedure types in men from more deprived areas, although the magnitude of the increased hazard diminished with increasing age.

In women, population-based ratios of repair rates in the most relative to the least deprived areas were generally higher than the corresponding ratios for men in all procedure categories and age groups. However, the reduction in rate ratios with increasing age was less consistent and less marked. Patterns of association between socioeconomic deprivation, procedure types, and survival after surgery were less clear in women.

There was evidence of socioeconomic disparities in population-based AAA repair rates in men, which was particularly evident for elective repair. The national screening programme in England^5^ found that the prevalence of AAA in men aged 65 years was 80 per cent higher in the most, relative to the least, deprived areas by quintile. However, the elective repair rates, which were 41–60 per cent higher in the 55–64-year age group and 0–33 per cent higher in the 65–74-year age group, for open and endovascular repair respectively, were not as high as might be expected given the underlying prevalence. Elective repair rates in the groups aged 75–84 and 85 or more years were in fact 5–26 per cent lower in the most deprived areas, although imprecision led to uncertainty in some of these estimates. One potential explanation is that elective AAA repair is less likely to be considered for elderly people from more deprived areas because of a higher prevalence of co-morbidities; however, the population-based rates could not be adjusted for co-morbidities. Another potential explanation is that the underlying prevalence of AAA eventually becomes lower in very elderly people living in these areas as a result of the selective survival hypothesis, a phenomenon that has been described in relation to ethnicity^36^, and also stroke^37,38^. In this scenario, the lower repair rates among elderly people in more deprived areas would be expected. However, there is little in the way of robust data on prevalence of AAA by deprivation in elderly people.

Another key new finding is that the population-based non-intervention rate was higher (ranging from 12 to 190 per cent higher) in men and women admitted from more socioeconomically disadvantaged areas. This could be a reflection of the higher population prevalence of AAA in more disadvantaged areas^5^. However, the association is reflected in the results when examined as the likelihood of death without aneurysm repair in admitted men. One potential explanation is that patients from more deprived areas are more unwell with greater co-morbidities resulting in them being considered unlikely to be able to withstand major surgery. However, adjustment for co-morbidities made little difference to the higher ORs for death without aneurysm repair in admitted patients.

With regard to mode of presentation, the finding that, among patients who had aneurysm repair, those from more socioeconomically deprived areas were more likely to have this performed for a ruptured AAA than patients from less deprived areas, is consistent with previous studies^10,14–16^. Although one previous study^17^ found that socioeconomically disadvantaged patients were more likely to have emergency rather than elective repair, and another did not^18^, the present study found clear evidence that, among patients having repair of intact AAA, those from more disadvantaged areas were more likely to have this done as an emergency procedure than those from less disadvantaged areas.

Previous studies comparing the likelihood of having open surgery or EVAR for AAA reported mixed results. Some^18,21^ found that patients from more socioeconomically disadvantaged backgrounds were more likely to have open repair, whereas others^19,22^ noted no association, and a further study^23^ documented an increased likelihood of having EVAR. The present study found that, among men who had an elective repair, those from more deprived areas were more likely to have EVAR than those from less deprived areas, although this was clearly seen only in the 65–74- and 75–84-year age groups.

With regard to socioeconomic disparities in survival after AAA repair, several^10,14,22,24–26^, although not all^16,18,23,27^, studies reported worse survival in patients from socioeconomically disadvantaged backgrounds. The present study found clear evidence that survival after repair was worse in men from socioeconomically disadvantaged areas for all presentation and procedure types.

The observed socioeconomic disparities may be explained by higher levels of co-morbidities in patients from more deprived areas that were not adjusted for adequately in the analyses, and by lower uptake of AAA screening by men in more deprived areas^5^, resulting in late presentation. However, there may also be other more general potential explanations. These include disparities in access to healthcare^39^, disparities in referral decisions and navigation of the healthcare system^40^, and disparities in waiting time for elective surgery^41^. However, other research^42^ has suggested that more socially disadvantaged people tend to consume more healthcare because they are sicker. With regard to interaction with health professionals, there may be a social gradient in doctor–patient communication and shared decision making^43,44^, disparities in length of consultation, empathy, and patient-centred care^45^, and implicit (unconscious) bias^46^.

This study was able to carry out a comprehensive examination of the association between socioeconomic disadvantage and aneurysm repair rates and survival using a large national data set, which facilitated nuanced analyses in relation to age, sex, mode of presentation, and procedure type. Nevertheless, there are a number of limitations to be considered. All admissions are likely to have been captured by the NHS systems in place, but there may have been errors in coding leading to misclassification of operative procedures. There may also have been variation in coding over time.

The HES data set is essentially an administrative data set and contains limited clinical information; it lacks information on aneurysm size and whether patients have been screened previously for AAA. Although the analyses were adjusted for co-morbidities, the limited information on co-morbidities could have resulted in incomplete adjustment for higher prevalence of co-morbidities in patients living in disadvantaged areas.

LSOA-level population counts are estimates and may have overestimated or underestimated population counts. The measure of socioeconomic deprivation was at the area level as the NHS system does not collect individual-level data on socioeconomic status. Use of the IMD from a single year could potentially have led to misclassification of some LSOAs into an incorrect deprivation category at the extremes of the time interval examined.

Disparities have been observed in several branches of surgery. Two systematic reviews^47,48^ have proposed conceptual frameworks for classifying factors contributing to surgical disparities, including patient, provider, and system-level factors. A comprehensive review^49^ noted that, although there was a substantial body of literature on documenting, measuring, and understanding the causes of surgical disparities, the evidence base for rigorous evaluation of interventions to reduce disparities was very limited.

It is likely that factors influencing socioeconomic disparities in AAA repair rates and outcomes are similar to those affecting other conditions. However, each condition may also have some unique issues when considering how such disparities may be addressed. There are socioeconomic differences in the prevalence of AAA and uptake of screening^5^, which could lead to more deprived populations having a later diagnosis, greater risk of emergency surgery, and poorer outcomes.

This situation, however, presents a potential opportunity for addressing the disparities observed. Targeted interventions that increase the uptake of screening in deprived populations might both provide the opportunity to identify and treat AAA in groups with a higher incidence and risk, and also allow earlier identification of this cohort of patients, who may benefit from lifestyle and other interventions, while under AAA surveillance, to reduce operative risks and cardiovascular mortality.

## Supplementary Material

znac222_Supplementary_Data
