# Supplementary material for: Socioeconomic disparities in abdominal aortic aneurysm repair rates and survival
Source: Br J Surg. 2022 Aug 11;109(10):958–67. doi: 10.1093/bjs/znac222 (PMC10364757; doi:10.1093/bjs/znac222)
Supplement: znac222_Supplementary_Data [file znac222_supplementary_data.docx]

**Supplementary material**

***Population-based aneurysm repair rates***

Trends over time in procedure rates by age and sex are shown in Figure S1. Men had a striking increase in elective EVAR rates between 2006/07 and 2010/11 followed by a plateau and gradual decrease. This was accompanied by a striking decrease in elective open repair rates over the time period examined. In the 55-64 year age group in the most recent years, elective open rates remained marginally higher than elective EVAR rates. However, in the 75-84 and 85+ age groups, elective EVAR rates became substantially higher than elective open rates in the most recent years. There was a gradual decline in emergency non-ruptured repair rates over time in all age groups except the 85+ age group. Repair rates for ruptured AAA declined in all age groups although the trend was less consistent in the 85+ age group. Rates of death in hospital without AAA repair declined over time in all age groups examined.

In women, the trends in elective open and EVAR rates and emergency repair rates for intact aneurysms were broadly similar to those in men. The trends for repair of ruptured AAA were also broadly similar except in the 85+ group where the decline was less clear. Patterns in rates of death in hospital without AAA repair were also similar to those seen in men. The substantially higher rates of death in hospital without AAA repair relative to the repair rates for the different procedures in the 85+ age group declined over time but still remained clearly higher in the most recent years.

***Survival following aneurysm repair***

General patterns in survival curves following AAA repair by sex and type of procedure for all patients aged 55+ years combined are shown in Figure S2. Patterns in men and women were similar. Initial survival was higher in the elective EVAR group than the elective open group because of the sharp initial decrease in survival following open repair. Thereafter, the decrease in survival was less steep in the elective open group than the elective EVAR group, with survival curves crossing at around 1-2 years after surgery.

Within approximately the first eight years following surgery, survival rates were higher in patients undergoing elective repair than patients undergoing emergency repair for intact aneurysms. Beyond eight years after repair, there was no difference between survival rates in the elective EVAR and emergency non-ruptured groups.

Initial survival following surgery for ruptured AAA was much worse than initial survival in the other categories, with only around 50-60% of patients surviving the operation. After the initial sharp drop in survival, however, the gradient of decline in survival was relatively gentle.

***Hospitals providing AAA surgery within deprivation category areas***

Figure S3 shows the number of hospitals located within each deprivation category area that provided AAA repair over the 12-year study period. The number of hospitals within each deprivation category area decreased over the period examined. There was no gradient of association between socioeconomic deprivation and the number of hospitals located within each deprivation category area. Both the highest and lowest deprivation category areas generally contained the lowest number of hospitals providing AAA repair.

Figure S4 shows the percentage of hospitals providing AAA surgery within each deprivation category area that had an annual volume >=60 AAA repairs. The percentage of hospitals with an annual volume >=60 generally increased over the 12-year study time span in all deprivation category areas. There was no gradient of association between socioeconomic deprivation and the percentage of hospitals with an annual volume >=60. The most and least deprived category areas both generally had the highest percentage of hospitals with an annual volume >=60.

Table S1 shows the percentage of patients receiving elective AAA repair within each deprivation category who had their procedure performed at a hospital with an annual volume >=60. The percentage was generally similar across deprivation categories.

***In-hospital mortality, length of stay and readmissions following elective AAA repair***

Tables S2, S3 and S4 show the results of analyses examining the associations between socioeconomic deprivation and (i) in-hospital mortality within the same admission for AAA repair, (ii) length of stay, and (iii) readmissions within 30 days of discharge, for patients undergoing elective surgery.

The associations with socioeconomic deprivation were examined by (i) deprivation based on the deprivation category of the LSOA in which the patient lived, and (ii) deprivation based on the deprivation category of the LSOA in which the hospital where the patient’s AAA repair was performed was located. The statistical models included both variables to mutually adjust for the effects of each other, and also included adjustment for age, sex, type of repair, year and comorbidities.

Patients living in more socioeconomically deprived areas had higher odds of in-hospital death, longer lengths of stay and higher odds of readmission following elective AAA surgery compared with patients living in less deprived areas.

Patients treated in hospitals located in more deprived areas also had longer lengths of stay than patients treated in hospitals located in less deprived areas. However, there was no evidence of association between the deprivation level of the area in which the hospital was located and in-hospital mortality or readmissions following elective AAA surgery.

***Causes of death following aneurysm repair***

Table S5 shows the causes of death by deprivation category for patients with AAA who had aneurysm repair. The table includes deaths of patients operated on from 2007-2017 and which occurred at any time within this time frame. Around 22% of deaths had aortic aneurysm recorded as the cause of death with little variation across deprivation categories.

**Figure S1.** Annual time trends in operative procedure rates for AAA repair by age, sex and type of procedure in England from 2006/07 to 2017/18. Note that y-axis scales are different for all graphs due to the very wide range in rates.


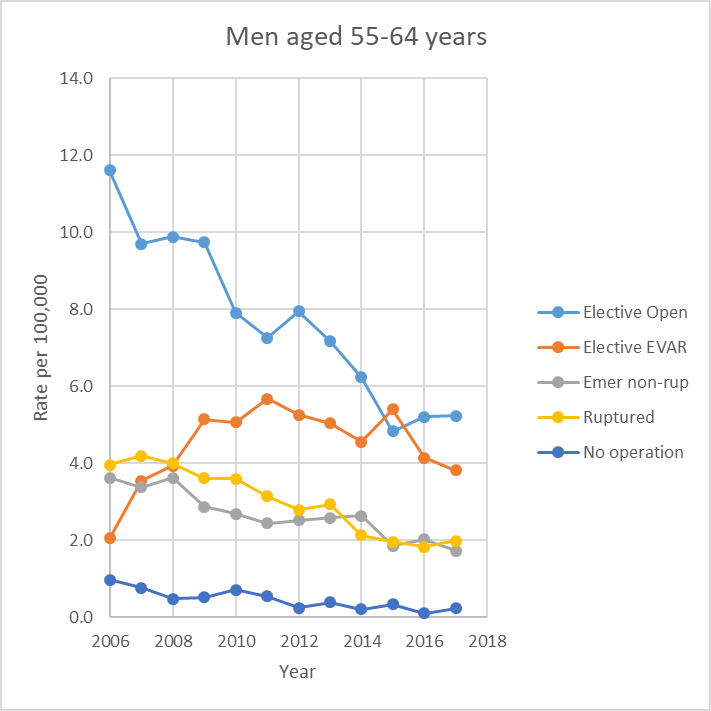

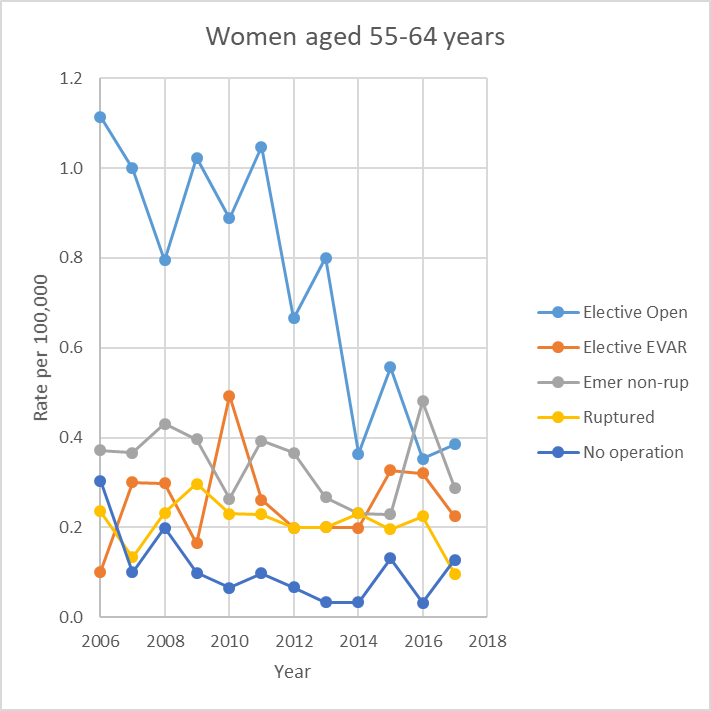


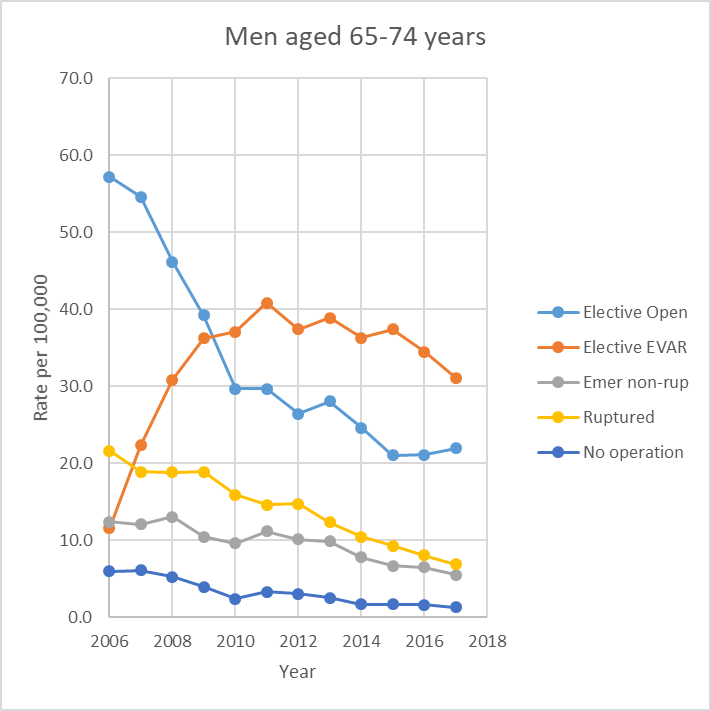

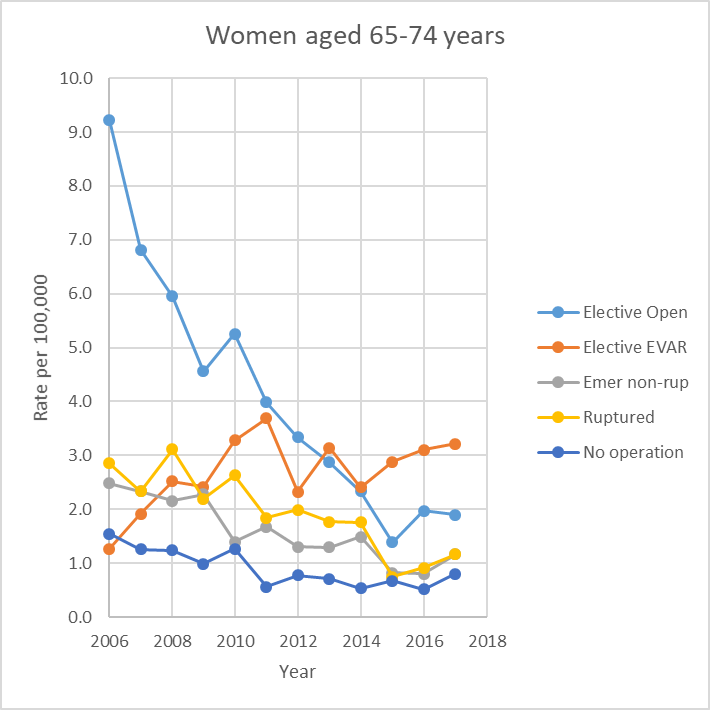


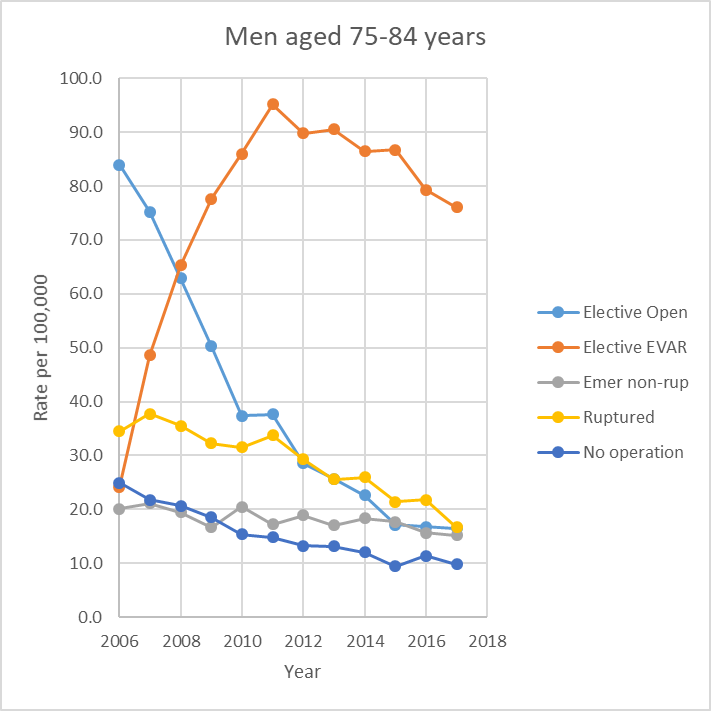

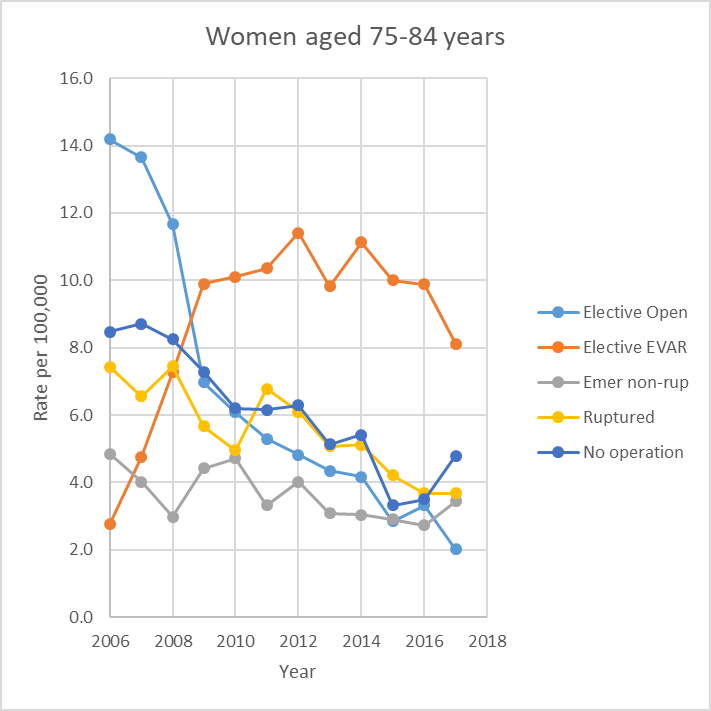


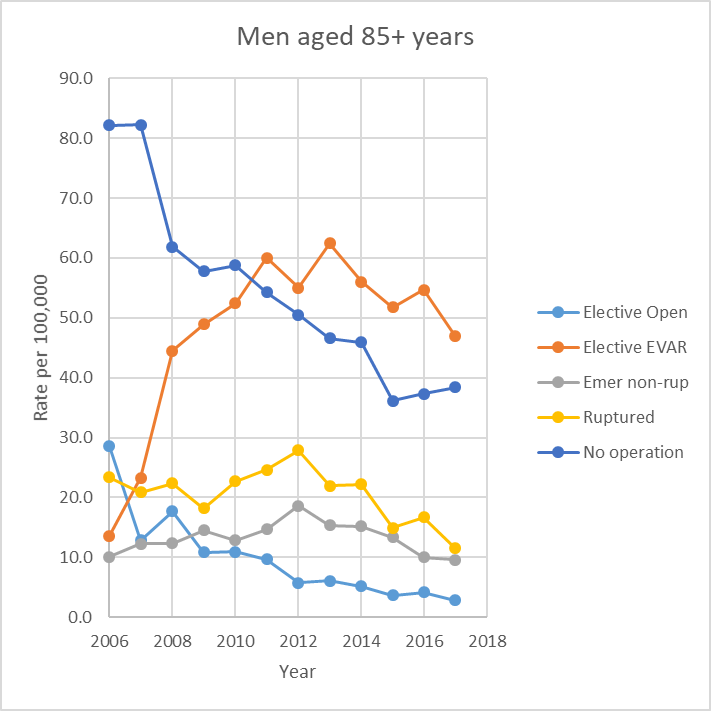

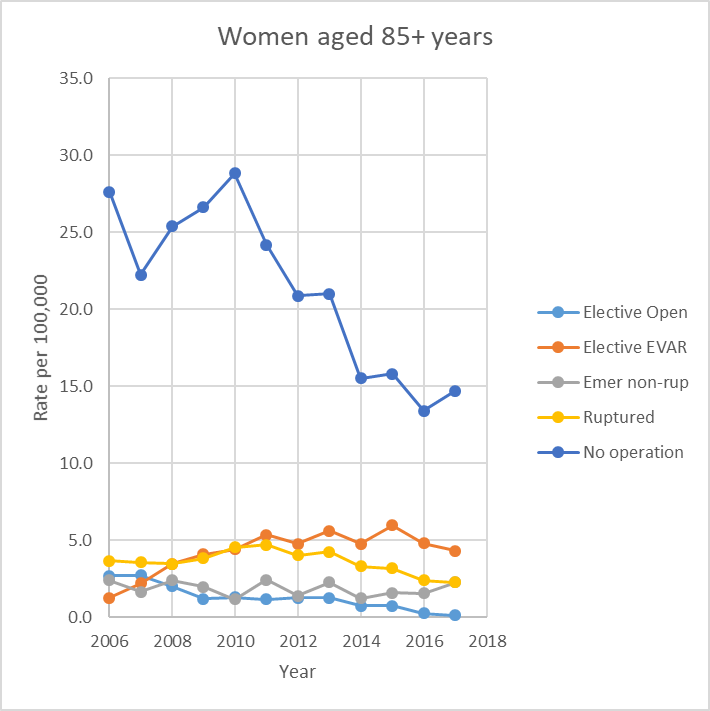


**Figure S2.** Survival following AAA repair by sex and type of procedure for all patients aged 55 years or more in England (April 2006 – March 2018).

(a) Men


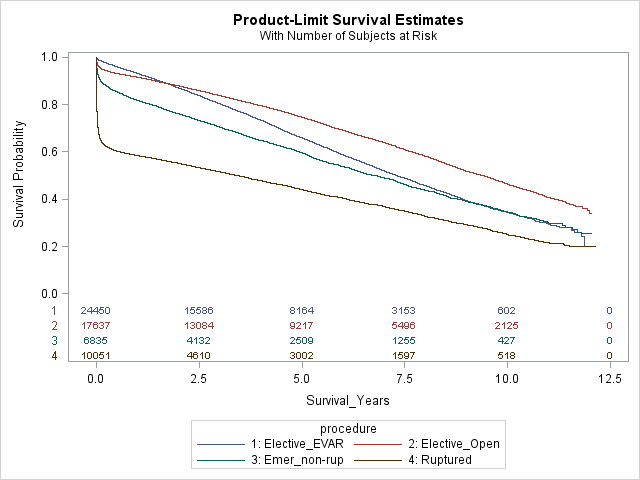


(b) Women


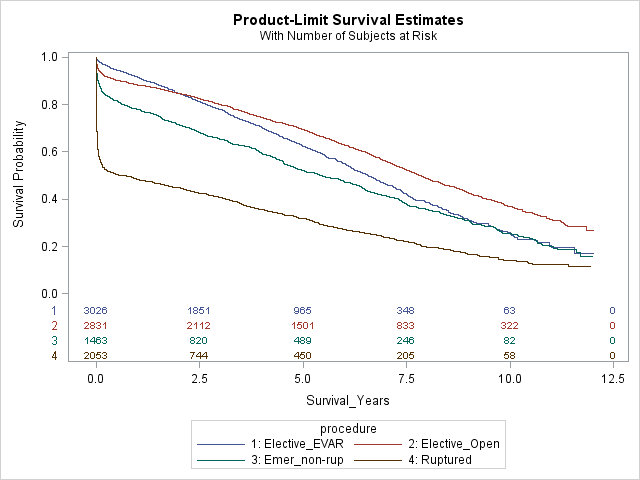


**Figure S3.** Number of hospitals providing AAA repair by location of hospitals within areas categorised by socioeconomic deprivation; England, 2006/07 to 2017/18.


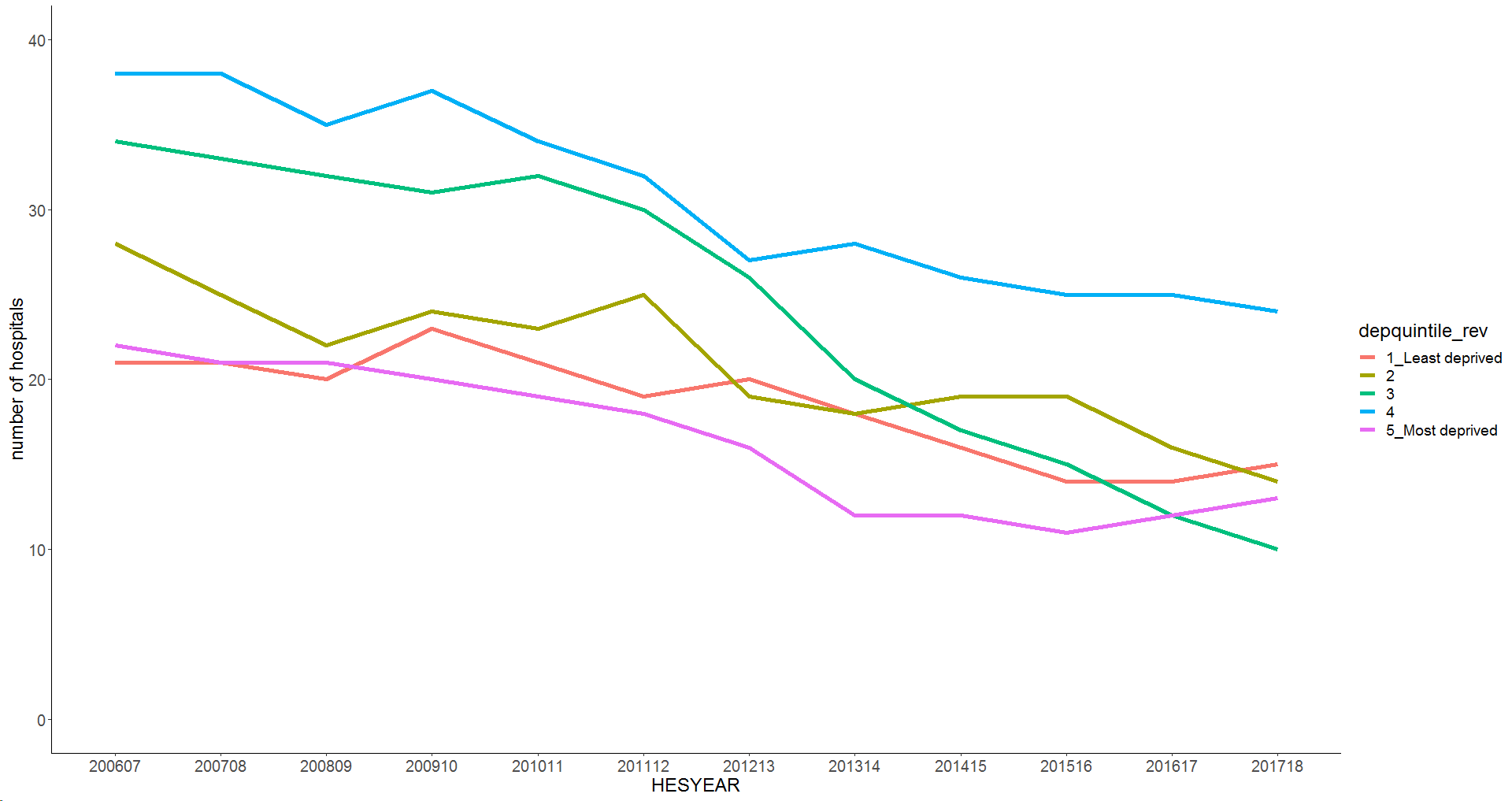


**Figure S4.** Percentage of hospitals providing AAA repair which had an annual volume of repair >=60 by location of hospitals within areas categorised by socioeconomic deprivation; England, 2006/07 to 2017/18.


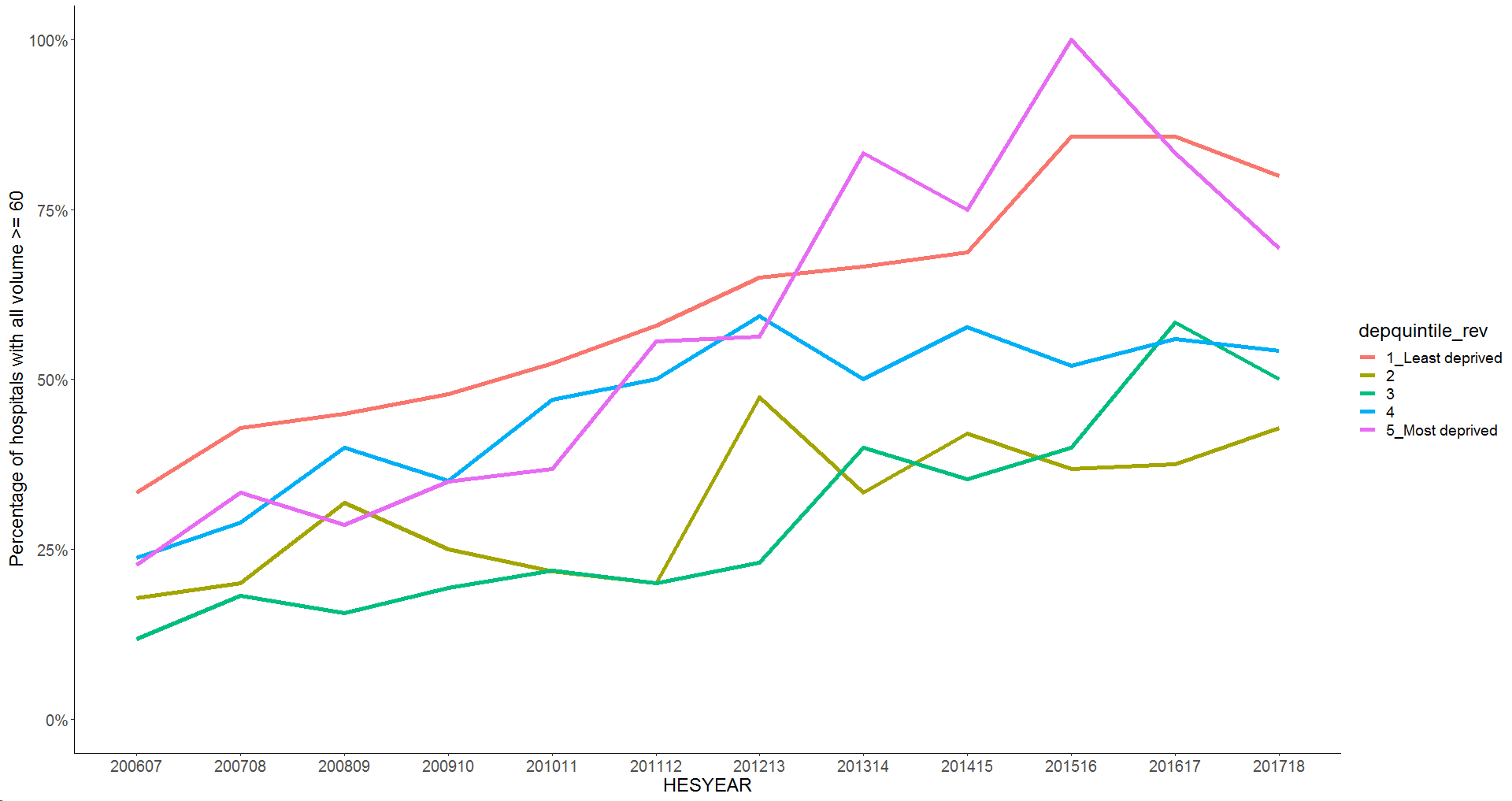


**Table S1.** Percentage of elective patients within each deprivation category who had their AAA repair performed at a hospital with an annual volume >=60 for AAA repair; England (April 2006 – March 2018).

| **Deprivation category** | **Average distance (miles) from patient residence to hospital** | **Percentage of cases performed at hospital with AAA surgery volume >= 60** |
| --- | --- | --- |
| 1 (least deprived) | 11.0 | 73% |
| 2 | 12.4 | 73% |
| 3 | 12.6 | 71% |
| 4 | 10.8 | 70% |
| 5 (most deprived) | 7.8 | 72% |

**Table S2.** Odds ratios (95% Confidence Interval) for in-hospital mortality within the same admission for patients who had elective surgery (open repair or EVAR); England (April 2006 – March 2018).

| **In-Hospital Death** | | | | |
| --- | --- | --- | --- | --- |
| **Variable*** | **Odds Ratio** | **Lower 95%CI** | **Upper 95%CI** | **P value** |
| EVAR | 0.18 | 0.16 | 0.21 | <0.001 |
| Men | 0.73 | 0.64 | 0.84 | <0.001 |
| Age (y) | 1.07 | 1.06 | 1.07 | <0.001 |
| HES Year 2007/08 | 1.07 | 0.88 | 1.32 | 0.491 |
| HES Year 2008/09 | 1.09 | 0.89 | 1.34 | 0.406 |
| HES Year 2009/10 | 0.95 | 0.76 | 1.19 | 0.662 |
| HES Year 2010/11 | 0.84 | 0.66 | 1.06 | 0.143 |
| HES Year 2011/12 | 0.88 | 0.70 | 1.11 | 0.293 |
| HES Year 2012/13 | 0.76 | 0.59 | 0.97 | 0.030 |
| HES Year 2013/14 | 0.80 | 0.62 | 1.02 | 0.073 |
| HES Year 2014/15 | 0.67 | 0.52 | 0.88 | 0.004 |
| HES Year 2015/16 | 0.70 | 0.53 | 0.92 | 0.011 |
| HES Year 2016/17 | 0.52 | 0.38 | 0.70 | <0.001 |
| HES Year 2017/18 | 0.65 | 0.49 | 0.86 | 0.003 |
| Coronary Artery Disease | 1.31 | 1.16 | 1.47 | <0.001 |
| Heart Failure | 1.27 | 1.03 | 1.57 | 0.029 |
| Cerebrovascular Disease | 0.98 | 0.76 | 1.25 | 0.855 |
| COPD | 1.46 | 1.30 | 1.64 | <0.001 |
| Diabetes | 1.13 | 0.97 | 1.30 | 0.115 |
| Renal Disease | 1.48 | 1.23 | 1.79 | <0.001 |
| Cancer | 1.09 | 0.92 | 1.28 | 0.330 |
| Moderate/Severe Liver Disease | 26.87 | 19.05 | 37.90 | <0.001 |
| Deprivation (Patient Residence) | 1.46 | 1.25 | 1.70 | <0.001 |
| Deprivation (Hospital Location) | 1.08 | 0.92 | 1.27 | 0.325 |

*** Interpretation of effects of variables**

For socioeconomic deprivation, the odds ratio is for the most relative to the least socioeconomically deprived quintile category. Odds ratios were calculated as a trend across all quintile categories and expressed as the ratio for the most relative to the least deprived category.

The patient residence deprivation is based on the deprivation category of the LSOA in which the patient lived.

The hospital location deprivation is based on the deprivation category of the LSOA in which the hospital where the patient’s AAA repair was performed was located.

EVAR is compared with Open Repair as the baseline.

Men are compared with Women as the baseline.

For age, the odds ratio is for an increase in age of one year.

HES Year 2006/07 is the baseline for comparison with other HES Years.

For comorbidities, the baseline is no comorbidity for each of the variables.

**Table S3.** Relative change (95% Confidence Interval) in length of stay for patients who had elective surgery (open repair or EVAR); England (April 2006 – March 2018).

| **Length of stay** | | | | |
| --- | --- | --- | --- | --- |
| **Variable*** | **Relative Change (multiplier)** | **Lower 95%CI** | **Upper 95%CI** | **P value** |
| EVAR | 0.39 | 0.38 | 0.40 | <0.001 |
| Men | 0.80 | 0.76 | 0.84 | <0.001 |
| Age (y) | 1.02 | 1.01 | 1.02 | <0.001 |
| HES Year 2007/08 | 0.96 | 0.89 | 1.03 | 0.273 |
| HES Year 2008/09 | 1.05 | 0.98 | 1.13 | 0.166 |
| HES Year 2009/10 | 1.00 | 0.93 | 1.07 | 0.926 |
| HES Year 2010/11 | 0.94 | 0.87 | 1.01 | 0.086 |
| HES Year 2011/12 | 0.97 | 0.90 | 1.04 | 0.405 |
| HES Year 2012/13 | 0.91 | 0.85 | 0.98 | 0.013 |
| HES Year 2013/14 | 0.85 | 0.79 | 0.91 | <0.001 |
| HES Year 2014/15 | 0.78 | 0.73 | 0.84 | <0.001 |
| HES Year 2015/16 | 0.77 | 0.71 | 0.83 | <0.001 |
| HES Year 2016/17 | 0.71 | 0.66 | 0.76 | <0.001 |
| HES Year 2017/18 | 0.64 | 0.59 | 0.69 | <0.001 |
| Coronary Artery Disease | 1.07 | 1.03 | 1.10 | <0.001 |
| Heart Failure | 1.14 | 1.07 | 1.22 | <0.001 |
| Cerebrovascular Disease | 1.19 | 1.11 | 1.28 | <0.001 |
| COPD | 1.16 | 1.12 | 1.20 | <0.001 |
| Diabetes | 1.09 | 1.05 | 1.13 | <0.001 |
| Renal Disease | 1.21 | 1.14 | 1.28 | <0.001 |
| Cancer | 1.02 | 0.97 | 1.06 | 0.501 |
| Moderate/Severe Liver Disease | 1.66 | 1.23 | 2.22 | 0.001 |
| Deprivation (Patient Residence) | 1.13 | 1.08 | 1.18 | <0.001 |
| Deprivation (Hospital Location) | 1.10 | 1.06 | 1.15 | <0.001 |

*** Interpretation of effects of variables**

For socioeconomic deprivation, the relative change is for the most relative to the least socioeconomically deprived quintile category. Relative change was calculated as a trend across all quintile categories and expressed as the ratio for the most relative to the least deprived category.

The patient residence deprivation is based on the deprivation category of the LSOA in which the patient lived.

The hospital location deprivation is based on the deprivation category of the LSOA in which the hospital where the patient’s AAA repair was performed was located.

EVAR is compared with Open Repair as the baseline.

Men are compared with Women as the baseline.

For age, the relative change is for an increase in age of one year.

HES Year 2006/07 is the baseline for comparison with other HES Years.

For comorbidities, the baseline is no comorbidity for each of the variables.

**Table S4.** Odds ratios (95% Confidence Interval) for readmission within 30 days of discharge after elective surgery (open repair or EVAR); England (April 2006 – March 2018).

| **30-day readmission** | | | | |
| --- | --- | --- | --- | --- |
| **Variable*** | **Odds Ratio** | **Lower 95%CI** | **Upper 95%CI** | **P value** |
| EVAR | 1.25 | 1.18 | 1.33 | <0.001 |
| Men | 0.86 | 0.79 | 0.93 | <0.001 |
| Age (y) | 1.01 | 1.01 | 1.02 | <0.001 |
| HES Year 2007/08 | 0.90 | 0.79 | 1.03 | 0.138 |
| HES Year 2008/09 | 0.97 | 0.85 | 1.11 | 0.703 |
| HES Year 2009/10 | 0.90 | 0.79 | 1.03 | 0.135 |
| HES Year 2010/11 | 1.06 | 0.93 | 1.21 | 0.394 |
| HES Year 2011/12 | 0.81 | 0.71 | 0.93 | 0.003 |
| HES Year 2012/13 | 0.90 | 0.79 | 1.03 | 0.120 |
| HES Year 2013/14 | 0.85 | 0.74 | 0.97 | 0.018 |
| HES Year 2014/15 | 0.82 | 0.71 | 0.94 | 0.005 |
| HES Year 2015/16 | 0.79 | 0.69 | 0.91 | 0.001 |
| HES Year 2016/17 | 0.83 | 0.72 | 0.95 | 0.008 |
| HES Year 2017/18 | 0.75 | 0.65 | 0.87 | <0.001 |
| Coronary Artery Disease | 1.18 | 1.12 | 1.26 | <0.001 |
| Heart Failure | 1.19 | 1.07 | 1.33 | 0.001 |
| Cerebrovascular Disease | 1.16 | 1.03 | 1.30 | 0.012 |
| COPD | 1.28 | 1.20 | 1.36 | <0.001 |
| Diabetes | 1.03 | 0.96 | 1.11 | 0.396 |
| Renal Disease | 1.68 | 1.53 | 1.83 | <0.001 |
| Cancer | 1.56 | 1.45 | 1.68 | <0.001 |
| Moderate/Severe Liver Disease | 1.59 | 1.00 | 2.51 | 0.049 |
| Deprivation (Patient Residence) | 1.13 | 1.04 | 1.22 | 0.003 |
| Deprivation (Hospital Location) | 0.96 | 0.89 | 1.04 | 0.351 |

*** Interpretation of effects of variables**

For socioeconomic deprivation, the odds ratio is for the most relative to the least socioeconomically deprived quintile category. Odds ratios were calculated as a trend across all quintile categories and expressed as the ratio for the most relative to the least deprived category.

The patient residence deprivation is based on the deprivation category of the LSOA in which the patient lived.

The hospital location deprivation is based on the deprivation category of the LSOA in which the hospital where the patient’s AAA repair was performed was located.

EVAR is compared with Open Repair as the baseline.

Men are compared with Women as the baseline.

For age, the odds ratio is for an increase in age of one year.

HES Year 2006/07 is the baseline for comparison with other HES Years.

For comorbidities, the baseline is no comorbidity for each of the variables.

**Table S5.** Causes of death by socioeconomic deprivation category, in patients aged 55+ years undergoing AAA repair in England; January 2007 – December 2017.

| **Cause of death**  **(ICD-10)** | **Percentage of deaths by cause within each deprivation category** | | | | | |
| --- | --- | --- | --- | --- | --- | --- |
|  | ***1 (Least deprived)*** | ***2*** | ***3*** | ***4*** | ***5 (Most deprived)*** | ***All*** |
| Aortic aneurysm and dissection (I71) | 21 | 21 | 21 | 22 | 22 | 22 |
| Ischaemic heart disease  (I20-I25) | 15 | 14 | 14 | 13 | 15 | 14 |
| Cerebrovascular disease  (I60-I69) | 6 | 5 | 5 | 5 | 5 | 5 |
| Respiratory disease  (J00-J99) | 12 | 12 | 13 | 13 | 14 | 13 |
| Malignant neoplasms  (C00-C97) | 22 | 23 | 22 | 23 | 22 | 23 |
| Dementia  (F01, F03, G30) | 4 | 4 | 4 | 4 | 3 | 4 |
| Other | 21 | 20 | 21 | 20 | 19 | 20 |
| All | 100 | 100 | 100 | 100 | 100 | 100 |
| No. of deaths | 5036 | 5936 | 6068 | 5651 | 4866 | 27557 |
